# The Army Study to Assess Risk and Resilience in Servicemembers (Army STARRS): progress toward understanding suicide among soldiers

**DOI:** 10.1038/s41380-018-0197-z

**Published:** 2018-08-13

**Authors:** James A. Naifeh, Holly B. Herberman Mash, Murray B. Stein, Carol S. Fullerton, Ronald C. Kessler, Robert J. Ursano

**Affiliations:** 1 Department of Psychiatry, Center for the Study of Traumatic Stress, Uniformed Services University of the Health Sciences, Bethesda, MD USA; 2 Department of Psychiatry and Department of Family Medicine and Public Health, University of California San Diego, La Jolla, CA USA; 3 VA San Diego Healthcare System, San Diego, CA USA; 4 Department of Health Care Policy, Harvard Medical School, Boston, MA USA

**Keywords:** Diseases, Psychology, Genetics

## Abstract

Responding to an unprecedented increase in the suicide rate among soldiers, in 2008 the US Army and US National Institute of Mental Health funded the Army Study to Assess Risk and Resilience in Servicemembers (Army STARRS), a multicomponent epidemiological and neurobiological study of risk and resilience factors for suicidal thoughts and behaviors, and their psychopathological correlates among Army personnel. Using a combination of administrative records, representative surveys, computerized neurocognitive tests, and blood samples, Army STARRS and its longitudinal follow-up study (STARRS-LS) are designed to identify potentially actionable findings to inform the Army’s suicide prevention efforts. The current report presents a broad overview of Army STARRS and its findings to date on suicide deaths, attempts, and ideation, as well as other important outcomes that may increase suicide risk (e.g., mental disorders, sexual assault victimization). The findings highlight the complexity of environmental and genetic risk and protective factors in different settings and contexts, and the importance of life and career history in understanding suicidal thoughts and behaviors.

Since 2001, the US military has been engaged in the longest war in the Nation’s history. Army suicide rates, which were historically lower than the demographically-adjusted US general population, more than doubled between 2001 and 2009 (from 9.0 to 22.0 per 100,000) [1]. The US Army rate surpassed that of comparable civilians in 2008 and has remained elevated in the following years [2, 3]. In response, the Army in 2008 entered into an agreement with the US National Institute of Mental Health (NIMH) to seek a study to address this challenge. After a wide solicitation for proposals, the NIMH awarded funding of the Army Study to Assess Risk and Resilience in Servicemembers (Army STARRS, now called STARRS-LS for “longitudinal study”; www.starrs-ls.org), a multicomponent epidemiological and neurobiological study of risk and resilience factors for suicidal thoughts and behaviors (STBs), and their psychopathological correlates among Army personnel [4]. At the time, systematic and textured research on military STB was very limited.

Army STARRS (2009–2014) and STARRS-LS (2015–2020) are designed to identify modifiable risk and resilience factors, in order to better target interventions for Army suicides and to expand the scientific knowledge of psychosocial and neurobiological risk and resilience factors for STB and their psychopathological correlates. Here we present a broad overview of Army STARRS, with the aim of familiarizing readers with the study and its findings to date. Although the primary focus of this body of work is on STB outcomes, Army STARRS also sought to improve understanding of potential STB risk factors, including mental disorders, traumatic brain injury (TBI), and sexual assault, in order to provide a more comprehensive framework for addressing STB among soldiers and informing future research with military populations.

## Design of Army STARRS

**Fig. 1:**
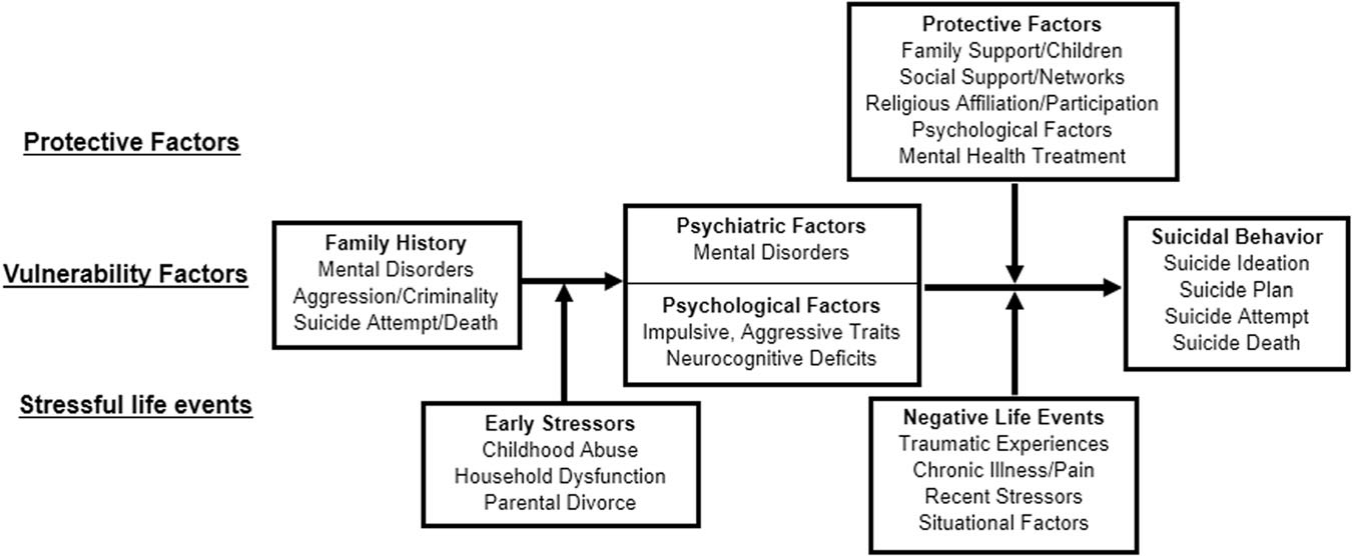
Vulnerability–stress model of suicidal behavior. (Reprinted with permission from Nock et al. [10])

Guided primarily by a vulnerability–stress perspective [5], the study was designed and conducted with consideration of various theoretical models of STB [6–9]. The investigators conducted preliminary literature reviews [10] and analyses of existing data [11–13] to inform and support Army STARRS. The research literature consistently asserts that STB develop through complex, multi-determined processes involving a large and diverse set of psychosocial and neurobiological factors. Suicide ideation, attempts, and deaths are distinct phenomena with different base rates, risk and protective factors, courses, treatment outcomes [14–18], and, presumably, genetic and neurobiological underpinnings [19, 20]. Conceptual models of STB commonly highlight the distinction between vulnerability factors and precipitating/initiating factors [21]. Precipitating/initiating factors are most often approached as stressors and adversities, with the types and timing of these events being key to identifying critical periods of suicide risk. Vulnerability factors or diatheses (e.g., family history of suicide, TBI, mental disorder) interact with stressors (e.g., traumatic events, loss of relationship, legal problems) and resilience factors (e.g., social support, religious participation, mental health treatment) to create psychological states of risk (e.g., hopelessness, feelings of burdensomeness, and entrapment) and social states of risk (e.g., detachment and lack of interpersonal contact). Suicidal behaviors can result when these risk states are coupled with available means and changes in individual psychology that allow for self-injury. Figure 1 provides an example of the vulnerability–stress perspective that underpins the approach of Army STARRS (described in detail elsewhere [10]).

Broadly, Army STARRS was designed to identify when, where, and for whom risk of STB is highest; identify modifiable risk and protective factors, including previously unknown factors; and develop predictive algorithms through concentration of risk models to target interventions in high-risk groups. Some of the specific research questions that guide the analyses include: (1) What are the sociodemographic, service-related, and mental health correlates of suicide ideation, attempt, and death, and how do those correlates vary across different environments and life/career phases (e.g., before enlistment, basic training, deployment)? (2) To what extent do deployment and combat exposure influence risk? (3) How does risk vary by time in service? (4) What factors predict the transition from thinking about suicide to acting on those thoughts? (5) What experiences do soldiers have before enlistment and how do those influence subsequent STB risk? (6) Can administrative and survey data be used to develop decision support tools to assist Army leaders, community service providers, and clinicians in targeting interventions and deploying resources? [22]

The Army STARRS team sought to balance the need for a multicomponent design and detailed assessment batteries with constraints on time available to access soldiers. Expert judgment was used to rank risk-resilience factors in order of expected importance, with particular attention to potentially modifiable factors. Pilot studies evaluated psychometric properties and developed short forms of selected instruments, and also adapted neurocognitive test batteries for group self-administration.

Army STARRS included a number of coordinated component studies (Table 1): (1) The Historical Administrative Data Study (HADS), which integrated 40 Army and Department of Defense (DoD) data systems containing administrative records for all 1.6 million soldiers on active duty from 2004 through 2009 (more than 1.1 billion records), including every data system in which suicide deaths, attempts, and ideation are administratively documented; (2) The New Soldiers Study (NSS), a representative survey of new soldiers in the first week of basic training with self-administered questionnaires (SAQs), computerized neurocognitive tests, and blood samples; (3) The All Army Study (AAS), a cross-sectional SAQ survey representative of active duty soldiers around the world (exclusive of basic training), including a probability sample of soldiers stationed in Afghanistan, who were surveyed as they transitioned through Kuwait for mid-tour leave; (4) The Pre-Post Deployment Study (PPDS), a four-wave panel survey of three brigade combat teams assessed with SAQs and blood samples just prior to deploying to Afghanistan and again at 1 month post-deployment, with additional SAQs administered at three and 9 months post-deployment; and (5) The Soldier Health Outcomes Studies (SHOS), two retrospective case–control studies of hospitalized suicide attempters (SHOS-A) and suicide decedents (SHOS-B), each including matched controls selected from AAS respondents. There was also a clinical reappraisal study to ensure diagnostic concordance between SAQ instruments and clinical interviews [23]. In addition to providing written informed consent for participation, respondents were asked to consent to linkage between their SAQ responses and Army/DoD administrative data. This enabled analysis of respondents’ Army career and medical records forward and backward in time from the date of their survey. Using personnel data provided by the Army, the samples in each study component were weighted to be representative of their target population. Additional information on the Army STARRS design, field and sampling procedures, weighting, neurocognitive testing battery, and psychometrics is available elsewhere [24–30]. In total, Army STARRS surveyed more than 100,000 soldiers. Its extensive databases allow investigation of a diverse combination of factors from demographic, psychological, biological, neurocognitive, behavioral, and social domains.

**Table 1:** Components of the Army STARRS

Army STARRS component	Sample size	Solders providing blood
HADS		
Analysis of 40 integrated Army/DoD administrative data systems with records for all soldiers on active duty during 2004–2009	> 1.6 million	NA
NSS		
Representative survey of soldiers in the first week of basic training	55,814	34,986*
AAS		
Representative survey of all soldiers around the world exclusive of those in basic training	41,210	NA
PPDS		
Four-wave panel survey of three brigade combat teams before and after deployment to Afghanistan	10,116	8090 at T0; 8822 at T1
SHOS-A		
Case–control study of hospitalized suicide attempters and matched soldiers from the AAS	186 Cases, 375 controls	296*
SHOS-B		
Case–control study based on interviews with the next-of-kin and army supervisors of suicide decedents and matched soldiers from the AAS	150 Cases, 270 controls	NA
Clinical Reappraisal Study		
Psychometric study examining concordance between DSM-IV diagnoses from self-administered questionnaires and diagnoses based on clinical interviews	460	NA

*Blood collection was added to the NSS and SHOS-A several months after data collection had already begun.

*AAS* All Army Study, *Army STARRS* Army Study to Assess Risk and Resilience in Servicemembers, *HADS* Historical Administrative Data Study, *NA* not applicable, *NSS* New Soldiers Study, *PPDS* Pre-Post Deployment Study, *SHOS-A* Soldier Health Outcomes Study A, *SHOS-B* Soldier Health Outcomes Study B

## Suicide death

### Historical Administrative Data Study

Army STARRS investigators conducted a systematic review of Army Criminal Investigation Division files for 510 suicides and 488 accident, homicide, or undetermined deaths during 2005–2009, to assess potential misclassification of non-suicide deaths. Only 1 of the 488 (0.2%) other deaths was reclassified as a definite suicide, suggesting that misclassification of suicides was uncommon [31].

**Fig. 2:**
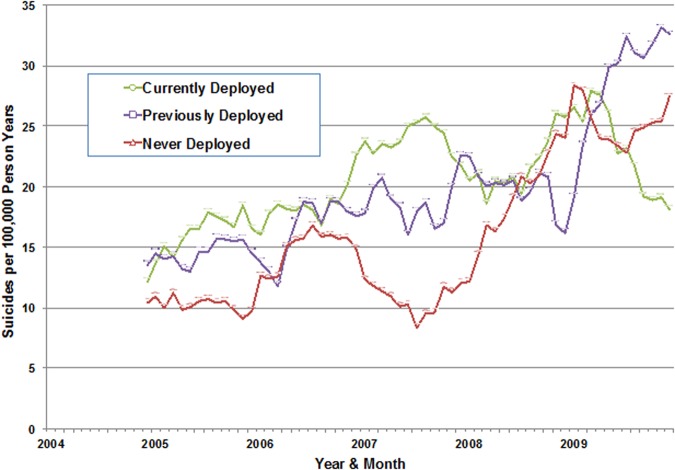
Suicide deaths per 100,000 person-years of active duty Army service among never deployed, currently deployed, and previously deployed Regular Army soldiers in the Army STARRS Historical Administrative Data Study (HADS), 2004–2009. Shown are Regular Army 12-month moving averages. Each line represents a 12-month moving average (i.e., each respective dot reports the rate for the prior 12-month period). (Reprinted with permission from Schoenbaum et al. [32])

The HADS provides an opportunity to examine STB risk using data that the Army routinely collects on all soldiers. Analysis of Army/DoD administrative data on all 569 suicides during 2004–2009 and a 1:400 equal-probability control sample of all other person-months demonstrated that the Regular Army suicide rate during this period increased among soldiers who were currently deployed (*N* = 140 suicides) and previously deployed (*N* = 236 suicides), as well as among those who were never deployed (*N* = 193 suicides) (Fig. 2) [32]. The time trend in suicide risk among never-deployed soldiers suggests that exposure to combat-related trauma was not the exclusive cause of the increase in Army suicides, although this requires further examination. Elevated risk was associated with male gender, white race/ethnicity, junior enlisted rank, recent demotion, and current or previous deployment. Neither the time trends in these predictors nor the Army’s increased use of accession waivers (relaxing some criteria for Army entry) explained the rise in Army suicides [32].

Using a career history approach that stratified the Army population by rank, time in service ( ≤ 4 years vs. > 4 years), and deployment status (never, currently, or previously deployed) revealed several novel findings [33]. Compared with the Army-wide suicide rate of 18.5/100,000 person-years (*N* = 569 suicides), rates were significantly elevated among junior enlisted soldiers who either deployed during their first year of service or had a lower rank than would be expected based on their time in service (69.6–80.0/100,000 person-years). The often-found protective effect of marriage was present only among deployed enlisted soldiers. Notably, there was a substantially greater relative rise in suicide among women than men during deployment. Several hypotheses that might explain the increased risk among deployed enlisted women were examined (e.g., gender proportions within occupations and units, prior sexual assault victimization, pre-deployment history of treated mental/behavioral disorders), but none could fully account for this disproportionately elevated suicide rate, highlighting the importance of expanding future research on the psychological challenges of deployment for women [34].

Among male enlisted soldiers (*N* = 496 suicides), two occupational categories (both in combat arms) had significantly elevated suicide rates compared with the overall rate for this subpopulation (22.4/100,000 person-years): infantrymen (37.2/100,000 person-years), and combat engineers (38.2/100,000 person-years) [35]. Interestingly, suicide rates in these two occupational categories were significantly lower when the soldiers were currently deployed (30.6/100,000 person-years) than when they were never deployed or previously deployed (41.2–39.1/100,000 person-years), whereas the suicide rate of other occupations was significantly higher when currently or previously deployed (20.2–22.4/100,000 person-years) than never deployed (14.5/100,000 person-years) [35].

### Soldier Health Outcomes Study - B

The SHOS-B psychological autopsy study provides intensive interview data from next-of-kin and Army supervisor informants for 135 suicide cases and two control groups selected from AAS participants: 137 propensity score-matched controls and 118 controls who reported past-year suicide ideation [36]. Most (79.3%) soldiers who died by suicide had a prior mental disorder. Mental disorders in the prior 30 days were especially strong risk factors and approximately half of suicide decedents told someone that they were considering suicide, suggesting opportunities for prevention and intervention. Although most risk factors differed between suicide cases and propensity-score-matched controls, they did not significantly differ between suicide cases and suicide ideators, pointing to an important direction for future research. The most striking difference between decedents and ideators was the presence in the former of an internalizing disorder (especially depression) and multi-morbidity (i.e., 3+ disorders) in the past 30 days [36].

## Suicide ideation and attempt

### Historical Administrative Data Study

Compared with suicide deaths, research on documented non-fatal suicidal behaviors was limited before Army STARRS. Suicide attempts, suicide ideation, and suspicious injuries (i.e., potential unrecognized suicide attempts) were identified using administrative medical records. There were 21,740 unique Regular Army soldiers who had at least one of these non-fatal outcomes documented during the 2004–2009 study period, with substantial increases in the annual incidence rates of suicide attempts (179–400/100,000 person-years; *N* = 885–2110 cases per year) and suicide ideation (557–830/100,000 person-years; *N* = 1930–3737 cases per year), but not suspicious injuries (55–60/100,000 person-years; *N* = 275–318 cases per year) [37].

**Fig. 3:**
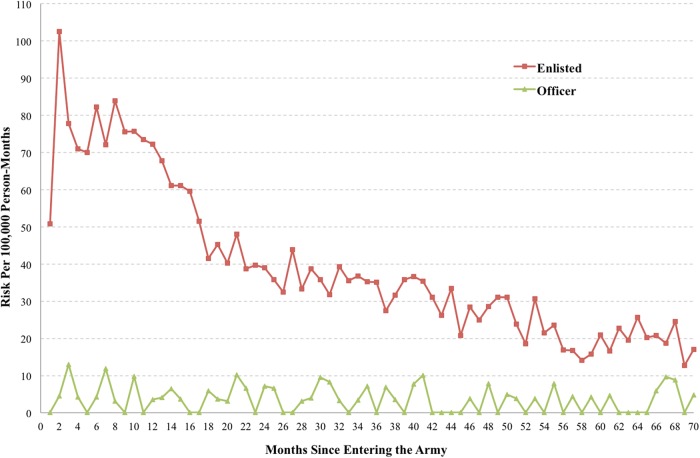
Risk of suicide attempt by month since entering service among Regular Army enlisted soldiers and officers in the Army STARRS Historical Administrative Data Study (HADS), 2004–2009. Discrete-time hazard functions were used to calculate risk estimates (suicide attempters per 100,000 person-months) for each month since entering Army service. (Reprinted with permission from Ursano et al. [38])

Enlisted soldiers accounted for 98.6% (*N* = 9650) of the 9791 Regular Army suicide attempters during 2004–2009, with an overall rate of 377/100,000 person-years. Officers accounted for the remaining 1.4% (*N* = 141) of attempters (27.9/100,000 person-years). Using a 1:200 equal-probability control sample of all other person-months, significant multivariable predictors among suicide attempt enlisted soldiers included sociodemographic (female gender, younger age, lower education, non-Hispanic White) and service-related (older age at Army entry, less time in service, being never or previously deployed [vs. currently deployed]) characteristics, and the presence and recency of mental health diagnosis. Among officers, only sociodemographic characteristics (female gender, older age at Army entry, younger current age, and low education) and the presence and recency of mental health diagnoses significantly predicted suicide attempt. Discrete-time hazard functions (Fig. 3) demonstrated that risk of suicide attempt among enlisted soldiers peaked around the second month of service, followed by a sharp decline that continued through the second year of service, with risk gradually leveling out toward the end of the first tour of duty. In contrast, risk among officers remained relatively stable over time [38]. Similar findings were observed when examining the multivariable predictors and patterns of medically documented suicide ideation (*N* = 10,466 cases), highlighting the need for research aimed at differentiating suicide-related outcomes of increasing severity [39].

**Fig. 4:**
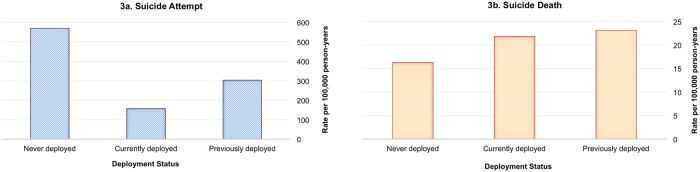
Crude rates of suicide attempt and suicide death by deployment status among Regular Army enlisted soldiers in the Army STARRS Historical Administrative Data Study (HADS), 2004–2009

**Fig. 5:**
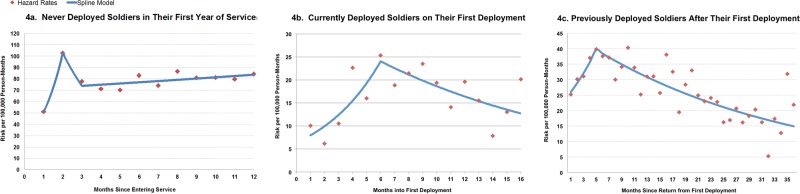
Monthly risk of suicide attempt risk by deployment status among Regular Army enlisted soldiers in the Army STARRS Historical Administrative Data Study (HADS), 2004–2009. The sample of enlisted soldiers (never deployed soldiers in their first year of service, *n* = 24,741; currently deployed soldiers on their first deployment, *n* = 13,833; and previously deployed soldiers after their first deployment, *n* = 38,281) is a subset of the total sample (*n* = 193,617 person-months) from the Army STARRS Historical Administrative Data Study (HADS). Monthly risk based on hazard rates and linear spline models. (Reprinted with permission from Ursano et al. [40])

Figure 4 demonstrates how crude rates of suicide attempt vary by deployment status and how that pattern differs from rates of suicide death. The 40% of enlisted soldiers who had never deployed accounted for 61% of enlisted suicide attempters (*N* = 5894; 569/100,000 person-years), whereas previously and currently deployed soldiers accounted for 29% (*N* = 2816; 304/100,000 person-years) and 10% (*N* = 940; 157/100,000 person-years) of attempters, respectively [40]. Regardless of deployment status, suicide attempts were more likely among soldiers who were female, in their first 2 years of service, or had recently received a mental health diagnosis. Odds of a suicide attempt increased with the number of prior mental health diagnoses, and the effect of multiple diagnoses was most pronounced among currently deployed soldiers. Risk was highest in the second month of service among those never deployed, the sixth month in-theater among those currently on their first deployment, and 5 months after returning home among those previously deployed (Fig. 5). Drug overdose was the most common method of suicide attempt and accounted for more than 50% of enlisted attempts in each deployment status. Although infrequent in non-fatal suicide attempts, the use of firearms was more likely to occur among currently deployed (21/100,000 person-years; *N* = 127 attempters) or previously deployed soldiers (14/100,000 person-years; *N* = 108 attempters), compared with soldiers who had never deployed (5/100,000 person-years; *N* = 56 attempters) [40].

The length of time in the Army prior to one’s first deployment and the length of time between deployments (i.e., dwell time) are important for understanding suicide attempt risk. Among enlisted soldiers who deployed twice, risk of suicide attempt was higher for those who deployed for the first time after a shorter time in the Army (i.e., 12 months or less of service versus those with longer service before deployment) (odds ratio (OR) = 2.0 [95% confidence interval (CI) = 1.6–2.4]) and those who had a dwell time of 6 months or less vs. longer (OR = 1.6 [95% CI = 1.2–2.0]) [41]. Length of time in service before a first deployment and dwell time are modifiable risk factors for suicide attempt, contingent on the operational requirements of the wartime environment.

A soldier’s military occupation, which relates to an array of expected stressors and protective training, is related to risk. The adjusted odds of attempting suicide were higher in combat arms soldiers (OR = 1.2 [95% CI = 1.1–1.2]) and combat medics (OR = 1.4 [95% CI = 1.3–1.5], but lower in special forces (OR = 0.3 [95% CI = 0.2–0.5]), compared to all other occupations [42]. Combat arms and combat medics had higher odds of suicide attempt than other occupations when never deployed and previously deployed, but not when currently deployed. In the first year of service, primarily a time of training, combat medics had higher odds of suicide attempt than both combat arms and other occupations (ORs = 1.4–1.5) [42].

The extensive use of improvised explosive devices (IEDs) by enemy forces in the Iraq and Afghanistan wars was unprecedented [43]. Controlling for sociodemographics, service-related characteristics, and indicators of operations tempo/intensity (soldiers deployed/redeployed per month, combat deaths and injuries per month), soldiers’ risk of suicide attempt increased with increasing numbers of IEDs, with an attempt being 26% more likely for each 1000 IED increase in monthly frequency. The association of IED frequency with suicide attempt was stronger for soldiers in their first 2 years of service vs. those with 3 or more years of service. Importantly, among soldiers in their first 2 years of service, the IED-suicide attempt association was constant regardless of deployment status, meaning that its effect was constant (i.e., even among those not deployed), whereas among soldiers with 3 or more years of service, the association was higher for those never deployed and currently deployed versus previously deployed. The findings suggest that the threat of new weapons may increase stress burden among soldiers, even among non-deployed soldiers with no direct exposure, and particularly among those with less Army experience [43].

Additional findings suggest that Army units with a history of suicide attempts may be important targets for preventive interventions. In models adjusted for sociodemographics, service-related characteristics, prior mental health diagnosis, unit size, and unit deaths from suicide, combat, and accidents, enlisted soldiers were more likely to attempt suicide if 1 or more suicide attempts occurred in their unit during the past year, with odds increasing as the number of unit attempts increased to 5 or more (ORs = 1.4–2.3) [44]. The association of previous unit suicide attempts with subsequent risk was significant whether soldiers had a combat arms occupation or other occupation (OR = 1.4–2.3) and regardless of unit size, with the highest risk among those in smaller units (1–40 soldiers) (ORs = 2.1–5.9).

Violence within a soldier’s family (i.e., intrafamilial spousal or child violence/maltreatment) was also associated with risk of suicide attempt. Adjusted odds of suicide attempt were higher in soldiers with a history of family violence documented in legal, Family Services, and medical records (OR = 2.9 [95% CI = 2.6–3.2]) [45]. Risk increased as the number and recency of family violence incidents increased. Soldiers with past-month family violence were almost five times as likely to attempt suicide as those with no family violence history. Importantly, odds of attempt were elevated for both perpetrators (OR = 3.3 [95% CI = 3.0–3.7]) and victims (OR = 2.1 [95% CI = 1.8–2.5]) [45].

The HADS contains performance data from a computerized neurocognitive testing battery that is completed by all soldiers before deployment and provides baseline data in the event of TBI. Poorer neurocognitive test performance was prospectively associated with suicide attempt, even after controlling for sociodemographics and prior mental health diagnosis [46]. Additional analyses found similar prospective associations with documented suicide ideation and suicide death, suggesting that objective neurocognitive tests may enhance understanding and prediction of suicide risk [46].

### All Army Study

Unlike the HADS, which focuses on medically documented STB, the AAS provides self-report data from a representative sample of the Army population. In early analysis of the first 5428 AAS respondents (representative of non-deployed soldiers), lifetime prevalence estimates of self-reported suicide ideation, plans, and attempts were 13.9%, 5.3%, and 2.4%, respectively [47]. Most reported cases of STB (47.0%–58.2%) had pre-enlistment onsets. Most reported onsets of plans and attempts among ideators (58.3%–63.3%) occur within the year of onset of ideation. Approximately one-third of post-enlistment suicide attempts were associated with pre-enlistment mental disorders, suggesting that pre-enlistment mental disorders might be targets for early screening and intervention [47].

Subsequent analyses using data from a larger sample (the Consolidated AAS (*N* = 29,982), which includes the full AAS sample and respondents from the pre-deployment survey of the PPDS), found that prevalence estimates for lifetime suicide ideation were 12.7% among men and 20.1% among women, and for lifetime suicide attempts were 2.5% and 5.1%, respectively [48]. Consistent with the initial analysis of AAS respondents, retrospective age-of-onset reports indicated that most (53.4%–70.0%) of these outcomes had onsets before soldiers enlisted in the Army. For both men and women, being in the Regular Army (vs. National Guard or Army Reserve) and being in an enlisted rank (versus officer) was associated with increased risk of lifetime suicidal behaviors, which was present both before and after joining the Army [48]. For both genders, all self-reported lifetime mental disorders were associated with subsequent onset of suicide ideation. Among men, intermittent explosive disorder, panic disorder, and substance disorders (each characterized by agitation and impulsiveness) predicted the transition from suicide ideation to attempt [49].

Given the importance of the transition from suicide ideation to attempt, particularly in clinical settings where suicide risk is often assessed among patients with ideation, we used the Consolidated AAS sample to examine whether characteristics of suicidal thoughts predicted subsequent suicide attempts among lifetime ideators. In models adjusted for previously known predictors (e.g., demographics, mental disorders), the most powerful predictors of first lifetime suicide attempt were recent onset of ideation, presence and recent onset of a suicide plan, low controllability of suicidal thoughts, extreme risk-taking or “tempting fate,” and failure to answer questions about the characteristics of one’s suicidal thoughts. A predictive model using these risk factors had strong accuracy (area under the curve = 0.93), with 66.2% of all incident suicide attempts occurring among the 5% of soldiers with highest composite predicted risk. This high concentration of risk suggests that a useful model to support clinical decision-making could be constructed using these variables [50].

### New Soldiers Study

The NSS results highlight the importance of pre-military health and experiences. Among new soldiers (*N* = 38,507), lifetime prevalence estimates of pre-enlistment suicide ideation, plans, and attempts were 14.1%, 2.3%, and 1.9%, respectively [51], consistent with the retrospective reports of pre-enlistment prevalence from soldiers in the AAS. Importantly, and also consistent with AAS findings, most reported onsets of pre-enlistment suicide plans and attempts among new soldiers (73.3–81.5%) occurred within the first year after onset of ideation, underlining the relatively rapid transition from ideation to attempt. Given that prior suicidal behaviors are among the strongest predictors of later suicides, these NSS results suggest that consideration should be given to developing methods of obtaining valid reports of pre-enlistment STB from new soldiers to facilitate intervention targeting [51].

Most but not all new soldiers with a pre-enlistment history of suicide attempt reported a prior mental disorder (59.0%) [52]. Each disorder was associated with increased odds of pre-enlistment suicidal behavior. The problem of unplanned attempts is a particularly difficult challenge for clinical care. Only posttraumatic stress disorder (PTSD) and disorders characterized by irritability and impulsive/aggressive behavior (i.e., bipolar disorder, conduct disorder, oppositional defiant disorder, and attention-deficit/hyperactivity disorder) predicted unplanned attempts among ideators [52].

Understanding the cumulative role of stressful and traumatic life events on suicide risk is a critical component to identifying and better targeting those at risk. Nearly 1 in 5 new soldiers reported experiencing childhood maltreatment, which was strongly associated with lifetime suicidal behavior, even after adjusting for intervening mental disorders [53]. Importantly, among soldiers with lifetime ideation, certain maltreatment profiles were associated with elevated odds of subsequently planning and/or attempting suicide. More frequent physical assault/theft by peers during childhood was also associated with increased odds of lifetime suicide ideation and attempt, and being the victim of more frequent bullying comments/behaviors in childhood was associated with increased risk of ideation, plan, attempt, and, importantly, the onset of plans among ideators (i.e., progression from suicide ideation to suicide plan). Exposure to the most persistent bullying was associated with a two- to four-fold increased risk for suicidal behaviors [54].

### Genetic risk factors

We conducted genome-wide association studies (GWAS) of lifetime suicide attempts in the population-based cohorts from the NSS and PPDS (total *N* = 608 cases and *N* = 16,657 control subjects) and the clinical case–control sample of recent suicide attempters from SHOS-A (*N* = 51 cases and *N* = 112 control subjects) [55]. Meta-analysis of the European ancestry discovery samples revealed a genome-wide significant locus in association with suicide attempt near *MRAP2* (melanocortin 2 receptor accessory protein 2) and CEP162 (centrosomal protein 162) with 12 genome-wide significant SNPs in the region; peak single nucleotide polymorphism (SNP) rs12524136-T (OR = 2.88, *p* = 5.24 × 10^–10^). These findings were not replicated in the European ancestry subsamples of the replication or SHOS-A samples. However, the association of the peak SNP remained significant in a meta-analysis of all studies and ancestral subgroups (OR = 2.18 [95% CI = 1.70–2.80]). *MRAP2* is expressed in brain and adrenal cortex and is a plausible susceptibility gene for STB. Polygenic risk score (PRS) analyses showed some association of suicide attempt with bipolar disorder [55].

## Mental disorders and other adverse outcomes

### Mental disorders

In support of its vulnerability–stress perspective, Army STARRS also examined the prevalence and correlates of STB risk factors. Improved understanding of the vulnerabilities and stressors associated with STB can assist in identifying specific groups or mechanisms for interventions that could ultimately prevent suicides. Analysis of the first 5428 AAS respondents, representing the total non-deployed Army from Q2–4 2011, found that 25.1% met criteria for any past 30-day disorder (15.0% internalizing; 18.4% externalizing) and 11.1% for multiple disorders [56]. More than 76% of those with a mental disorder reported pre-enlistment onset of at least one 30-day disorder (49.6% internalizing; 81.7% externalizing). Severe role impairment was reported by 12.8% of soldiers. Controlling for sociodemographic and Army career characteristics, 30-day disorders with pre-enlistment and post-enlistment ages at onset both significantly predicted severe role impairment. Pre-enlistment disorders were more consistently powerful predictors than post-enlistment disorders [56].

Among new soldiers entering the Army (i.e., NSS), the lifetime prevalence of having any self-reported mental disorder (38.7%), an internalizing disorder (19.8%), or an externalizing disorder (31.8%) did not differ significantly with matched civilians, although three specific disorders (generalized anxiety, PTSD, and conduct disorders) and multi-morbidity were significantly more common among new soldiers than civilians [57]. In a test of stress sensitization theory, which hypothesizes that individuals exposed to childhood adversity will be more vulnerable to mental disorders from proximal stressors, we found that soldiers who had been exposed to childhood maltreatment were at an increased risk of past 30-day major depressive episode or generalized anxiety disorder following recent stressful experiences [58]. Identification of this potential vulnerability and improved stress management may be particularly important in military populations, in which servicemembers experience an abundance of unique stressors.

Also among new soldiers just entering the Army, the prevalence of lifetime (pre-enlistment) binge drinking was 27.2% among males and 18.9% among females, and respective estimates for heavy drinking were 13.9% and 9.4%. Among those with no or minimal drug use, 9.5% of males and 7.2% of females had lifetime alcohol use disorder (AUD). Relative to no alcohol misuse, binge drinking, heavy drinking, and AUD were associated with increased odds of all assessed mental disorders and other adverse outcomes (lifetime and past 30-day suicide ideation, probable lifetime TBI, and past-year motor vehicle accident). Strong bidirectional associations between alcohol misuse and mental disorders were observed, whereby prior mental disorders and suicide ideation were associated with onset of AUD and prior AUD was associated with onset of mental disorders and suicide ideation [59].

In an analysis designed to discover genetic loci associated with lifetime DSM-IV PTSD risk, two coordinated GWAS were performed on 3167 soldiers with lifetime PTSD and 4607 trauma-exposed controls from the NSS, and 947 lifetime PTSD cases and 4969 trauma-exposed controls from the PPDS [60]. Two statistically significant genetic variants were associated with PTSD among NSS soldiers. One variant, in samples from African American soldiers with PTSD, was in a gene (ANKRD55) on chromosome 5. In prior research, this gene has been found to be associated with various autoimmune and inflammatory disorders, including multiple sclerosis, type 2 diabetes, celiac disease, and rheumatoid arthritis. There were no significant genetic correlations observed between PTSD and six mental disorders and nine immune-related disorders. However, there was significant evidence of pleiotropy for PTSD and rheumatoid arthritis, and, to a lesser extent, psoriasis. Further efforts are needed to replicate the genome-wide significant association researchers found with the gene ANKRD55, and to clarify the nature of the genetic overlap observed between PTSD and rheumatoid arthritis and psoriasis [60]. Results from this study are currently being shared with the Psychiatric Genomic Consortium PTSD Group [61] and will become part of future meta-analyses.

Army STARRS investigators conducted the first-ever GWAS of social anxiety, an important behavior of distancing/avoiding social contact and possible support, within European American, African American, and Latin American ancestral groups in the NSS and PPDS, and then meta-analyzed across studies [62]. SNP-based heritability for social anxiety was significant (*h*^2^_g_ = 0.12, *p* = 2.17 × 10^−4^ in the European American group). One meta-analytically genome-wide significant locus was seen in each of the European American and African American groups. Social anxiety was genetically correlated (negatively) with extraversion but not with neuroticism or with an anxiety disorder factor score from external GWAS meta-analyses. These observations support a genetic contribution to social anxiety and, therefore, possibly lower social support, which is a risk factor for STB.

Traumatic life experiences are associated with alcohol use problems, an association that is likely to be moderated by genetic predisposition. To better understand these interactions, a gene-by-environment genome-wide interaction study (GEWIS) of alcohol use problems was conducted in NSS and PPDS soldiers (*N* = 16,361), as well as civilians from the Yale–Penn cohort (*N* = 8084) [63]. In African American subjects, an interaction of PRKG1 with trauma exposure in the STARRS cohort was identified and replicated in the Yale–Penn cohort. PRKG1 encodes cyclic GMP-dependent protein kinase 1, which is involved in learning, memory, and circadian rhythm regulation. This was the largest GEWIS performed in psychiatric genetics, and the first GEWIS examining risk for alcohol misuse, adding to a growing body of literature highlighting the dynamic impact of experience on individual genetic risk [63].

Given that sleep problems negatively impact mental health, physical health, and overall quality of life, and are associated with depression and suicide risk, Army STARRS conducted GWAS of insomnia disorder using the NSS and PPDS samples [64]. Results indicated that the genetic contributions to insomnia disorder were significantly positively correlated with major depressive disorder (*r*_g_ = 0.44, SE = 0.22, *p* = 0.047) and type 2 diabetes (*r*_g_ = 0.43, SE = 0.20, *p* = 0.037), and negatively with morningness chronotype (*r*_g_ = −0.34, SE = 0.17, *p* = 0.039) and subjective well-being (*r*_g_ = − 0.59, SE = 0.23, *p* = 0.009), indicating that insomnia associated loci may contribute to the genetic risk underlying a range of health conditions including psychiatric disorders and metabolic disease [64].

### Traumatic brain injury

Early examination of reported mild TBI in those entering the Army indicated that nearly half had a pre-enlistment concussion/mild TBI, highlighting this issue broadly for the US population [65]. Considering deployment, approximately 1 in 5 PPDS respondents reported experiencing a TBI (defined as loss or alteration of consciousness in association with a concussive blast or other injury) during their recent deployment (18.0% mild TBI, 1.2% more-than-mild TBI) [66]. Even after adjusting for other risk factors (e.g., pre-deployment mental health status, severity of deployment stress, prior TBI history), deployment-acquired TBI was associated with elevated odds of PTSD, generalized anxiety disorder, and major depressive episode [66]. In further analyses, persistent and severe post-concussive symptoms (PCS) were found among PPDS respondents who experienced a mild TBI, while deployed to Afghanistan [67]. Based on surveys administered at 1, 3, and 9 months post-deployment, soldiers with mild TBI were three times more likely to report PCS than other PPDS soldiers. More severe symptoms were reported by soldiers with a history of TBI or mental health problems (e.g., depression, anxiety, irritability) before the index deployment, or more severe deployment-related stress. Persistent and more severe symptoms were also more likely when there was TBI-related loss of consciousness or memory problems (vs. being “dazed” only). Female soldiers were more likely than males to experience poor recovery following mild TBI [67].

A cross-phenotype high-resolution PRS analysis of persistent PCS was conducted in 845 PPDS respondents who sustained TBI during deployment [68]. The PRS was derived from summary statistics of large GWAS of Alzheimer’s disease, Parkinson’s disease, schizophrenia, bipolar disorder, and major depressive disorder; and for years of schooling, college completion, childhood intelligence, infant head circumference, and adult intracranial volume. Although the study had more than 95% of statistical power to detect moderate-to-large effect sizes, no association was observed with neurodegenerative and psychiatric disorders, suggesting that persistent PCS do not share genetic components with these traits to a moderate-large degree. Subjects with a PRS associated with high infant head circumference recovered better from cognitive/emotional persistent PCS than other individuals. Enrichment analysis identified two significant gene ontology (GO) terms related to this result: GO:0050839~Cell adhesion molecule binding and GO:0050905~Neuromuscular process. The findings indicate that genetic predisposition to persistent PCS after TBI does not have substantial overlap with neurodegenerative and psychiatric diseases, but mechanisms related to early brain growth may be involved [68].

### Sexual assault

Among all female Regular Army soldiers with administratively recorded sexual assault victimization from 2004–2009 (*N* = 4238 cases) and 5:1 propensity score-matched controls with similar composite victimization risk (to control for non-random victimization exposure), women who were sexually assaulted had significantly elevated ORs of subsequent mental health treatment (OR = 2.5 [95% CI = 2.4–2.6]), PTSD treatment (OR = 6.3 [95% CI = 5.7–6.9]), suicide attempt (OR = 3.0 [95% CI = 2.5–3.6]), demotion (OR = 2.1 [95% CI = 1.9–2.3]), and attrition (OR = 1.2 [95% CI = 1.1–1.2]). Findings indicate that sexual assault victimization is associated with considerable distress and likely decreases force readiness [69].

### Accident death

Similar to suicide deaths, accident deaths are important and potentially preventable outcomes. From an Army population health perspective, accident deaths are the largest peace-time risk to life and their study can also aid in understanding suicide deaths by examining the degree to which risk factors for the two outcomes differ. Analysis of administrative data from 2004 to 2009 identified a total of 1331 accident deaths [32, 70]. Among enlisted soldiers, delayed rank progression or demotion and being male, unmarried, in a combat arms occupation, and of low rank/service length increased odds of accident death. Unique to officers was high risk associated with aviation specialties. Accident death risk decreased over the study period for currently deployed enlisted soldiers, whereas it increased over time for those never deployed. Mental health diagnosis was associated with accident death only for previously deployed and never-deployed enlisted soldiers. Models did not discriminate line-of-duty from not-line-of-duty accident deaths [70]. Based on these findings and the findings on suicide death reported above, the temporal patterns in accident and suicide mortality differed during 2004–2009, but their individual-level predictors were generally similar.

## Mental health service use

Understanding service use can target opportunities for care. Approximately 50% of suicide decedents from 2004 to 2009 accessed healthcare in the month before their death, with more than 25% accessing healthcare in the week prior to their death [71]. Compared to matched Army controls, mental health encounters were significantly more prevalent among soldiers who died by suicide. Suicide decedents who were male, never married, and non-Hispanic Black were less likely to access care before death. Administrative documentation of risk (prior STB) among suicide decedents was rare (13.8% during the previous 4 weeks; 24.5% during the previous 52 weeks); however, the research team did not have access to the complete electronic health record (e.g., narratives). Thus, although soldiers who died by suicide accessed mental healthcare, the results suggest that risk detection and prediction may be improved by enhancing administrative mechanisms of risk documentation [71].

Mental health service utilization and barriers to care are significant concerns in the Army’s efforts to prevent suicide and improve force health and readiness. Among 5428 AAS respondents, only 21.3% of soldiers who reported a past 30-day mental disorder indicated they were currently receiving mental health treatment [72]. Reported barriers to care were examined among AAS respondents with a current mental disorder who either did not seek treatment in the past year (*N* = 744) or discontinued treatment in the past year (*N* = 145) [73]. More than 82% of soldiers who did not initiate treatment and 69.5% of soldiers who discontinued treatment endorsed at least two barriers to care. Importantly, among those who did not initiate treatment, 69.8% reported no perceived need for mental health treatment. Attitudinal reasons (e.g., stigma, a desire to handle the problem on one’s own) were cited more frequently than structural reasons (e.g., lack of financial means, inconvenience) among both untreated soldiers with perceived need (80.7% vs. 62.7%) and soldiers who discontinued treatment (71.0% vs. 37.8%). The findings suggest most soldiers with mental disorders do not believe they need treatment and those who do typically face multiple attitudinal and, to a lesser extent, structural barriers [73].

## Machine learning and concentration of risk

In support of precision medicine approaches and cost-effective intervention targeting, Army STARRS investigators were among the first to demonstrate the potential utility of machine learning to assist clinical decision-making through enhanced risk prediction for adverse mental health and other outcomes. For example, the days, weeks, and months following discharge from psychiatric hospitalization are known to be a particularly high-risk period for suicide. During 2004–2009, 68 soldiers died by suicide within 12 months of hospital discharge (12.0% of all US Army suicides), equivalent to 263.9/100,000 person-years vs. the Army-wide rate of 18.5/100,000 person-years [74]. A total of 52.9% of post-hospitalization suicides occurred after the 5% of hospitalizations with highest predicted suicide risk based on machine learning methods (3824.1/100,000 person-years). These highest-risk hospitalizations also accounted for significantly elevated proportions of other adverse post-hospitalization outcomes (unintentional injury deaths, suicide attempts, and subsequent hospitalizations) [74].

Machine learning models have also been developed to predict the following: suicides after outpatient mental health specialty visits [75], sexual violence victimization [76] and perpetration [77], non-familial major physical violent crime perpetration [78], and minor violent crime perpetration [79], as well as diverse negative outcomes among new soldiers in the NSS [80]. The high concentration of risk obtained through these machine learning analyses suggests that such models can be useful in targeting soldiers with the highest need for preventive interventions, although final determination requires careful weighing of intervention costs, effectiveness, and competing risks. It is important to note, though, that models to predict which soldiers are at highest risk of negative outcomes are not the same as models to determine which soldiers would benefit most from preventive interventions. The latter models can only be estimated after interventions are put in place and it is possible to study the prescriptive predictors of differential intervention response. The STARRS-LS team is poised to carry out such analyses, and work along these lines is already underway as part of an outgrowth of STARRS-LS that involves implementing a preventive intervention for veterans estimated to be at high risk of suicide death [81].

## Conclusions and future directions

Army STARRS, called by some the Framingham of mental health studies [82], continues to generate potentially actionable findings to inform the Army’s suicide prevention efforts and explore areas of risk (e.g., suicide attempts, population-level estimates) not previously accessible in representative civilian populations. The Army will ultimately determine which findings are actionable by considering the costs and effectiveness of potential interventions within the evolving context of their mission and available resources. Overall, the sociodemographic predictors of STB among soldiers are generally consistent with those in the US general population [83, 84]. In both populations, suicide ideation and attempt are more likely among those who are female, younger, non-Hispanic White, and less educated. Suicide death in both populations is more likely among those who are male non-Hispanic White, and less educated. However, our results demonstrate that the associations of these predictors, as well as military-specific predictors (e.g., age at Army entry, rank, military occupation), often differ by deployment status and time in service, highlighting the complexity of understanding risk and protective factors in different contexts. As in the general population [85], most transitions from ideation to attempt among soldiers occur within 1 year of ideation onset [47, 48, 51]. Understanding and predicting the transition from thinking about suicide to acting is of critical importance and in need of more intensive study in all populations. Future research must also continue to examine how STB risk among current and former soldiers is influenced by pre-enlistment experiences (e.g., childhood maltreatment, history of STB, and history of mental disorders), and life and career transitions (e.g., entering Army service, returning from deployment, separating from the Army).

The work that began in Army STARRS continues in STARRS-LS (2015–2020), the longitudinal follow-up study funded by the DoD, which adds historical administrative data from 2010 to 2015 and reassessment of the original Army STARRS survey respondents via web- and telephone-based interviews. These follow-up surveys will capture soldiers who are still serving and those who have separated from the Army. The number of soldiers leaving the Army and returning to civilian life each year will increase as the Army downsizes with the end of the Afghanistan war. The transition from military to civilian life is known to be stressful [86, 87] and will presumably become more so with an increase in the number of soldiers who may want to remain in the Army but must instead return to civilian life. Army STARRS and now STARRS-LS provide a platform for studying trajectories of suicide risk and mental health over the life course of current and former soldiers. It also demonstrates the value of assessing new soldiers and using big data mental health studies to support precision medicine, which includes identifying populations at risk and developing decision support tools for clinicians and Army leaders. In doing so, the study offers valuable opportunities for understanding and mitigating suicide risk.
